# 5-Fluorouracil and actinomycin D lead to erythema multiforme drug eruption in chemotherapy of invasive mole: Case report and literature review

**DOI:** 10.1097/MD.0000000000031678

**Published:** 2022-11-25

**Authors:** Shan Wang, Tengfei Li, Yuan Wang, Mengdi Wang, Yibin Liu, Xiaoguang Zhang, Lijuan Zhang

**Affiliations:** a Departments of Gynecology, The Second Hospital of Hebei Medical University, Shijiazhuang, Hebei Province, China; b Departments of General Surgery, The Second Hospital of Lanzhou University, Lanzhou, Gansu Province, China; c Departments of Dermatology, The Second Hospital of Hebei Medical University, Shijiazhuang, Hebei Province, China.

**Keywords:** 5-Fluorouracil, actinomycin D, drug eruption, erythema multiform, side effects

## Abstract

5-Fluorouracil (5-FU) and actinomycin D (ActD) are often used in chemotherapy for various cancers. Side effects are more common in bone marrow suppression, liver function impairment, and gastrointestinal responses. Skin effects are rare and easy to be ignored by doctors and patients, which can lead to life-threatening consequence.

**Patient concerns:**

We reported a 45-year-old woman patient developed skin erythema and fingernail belt in chemotherapy of 5-FU and ActD.

**Diagnosis:**

Erythema multiforme drug eruption.

**Interventions:**

Laboratory tests including blood and urine routine, liver and kidney function, electrolytes and coagulation function and close observation.

**Outcomes:**

The rash was gone and the nail change returned.

**Lessons:**

Delays in diagnosis or treatment may lead to serious consequence. We should pay attention to the dosage of 5-FU and ActD, monitor adverse reactions strictly, to reduce occurrence of skin malignant events.

## 1. Introduction

5-Fluorouracil (5-FU) and its precursor drugs are fluoropyrimidine antineoplastic medications, which often used to treat cancer of the gastrointestinal, breast, ovarian, lung, cervical, bladder, skin and gestational trophoblastic neoplasia. As a broad-spectrum cell cycle-specific antimetabolic drug, it can kill proliferating cells, commonly used in clinical.^[1,2]^ Actinomycin D (ActD) is extracted from actinomycetes and many species of streptomyces, has good antitumor activity and plays an antitumor effect by inhibiting synthesis of nucleic acid and activity of some proteases, inducing cell differentiation and apoptosis.^[3,4]^ The most common side effects of 5-FU and ActD are bone marrow suppression, liver and kidney function damage and gastrointestinal reaction.^[5,6]^ The skin side effects of 5-FU are infrequent, including contact dermatitis, rosacea or seborrheic dermatitis, nail change, erythema of the extremities, actinic keratosis inflammation, pigmentation, etc.^[7]^ Skin toxicity of ActD is scarce. Although skin side effects are easy to be ignored by doctors and patients, some effect as Stevens-Johnson syndrome (SJS) and toxic epidermal necrolysis (TEN) may lead to serious consequences. In July 2021, a female patient with erosive mole was admitted in our hospital, in the 6th course of chemotherapy with 5-FU and ActD, erythema multiform was found in the skin, accompanied by nail changes.

## 2. Case report

**Figure 1.**
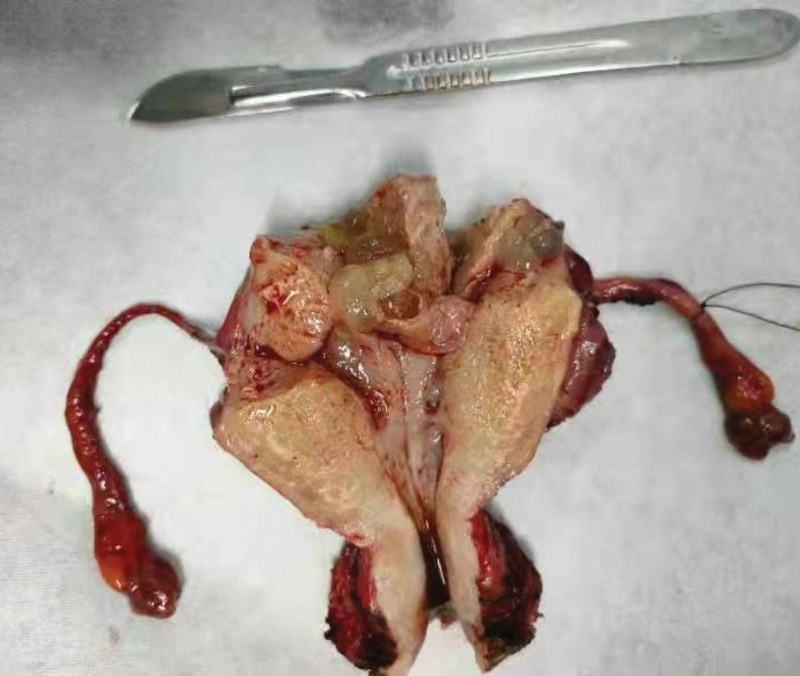
A macroscopic image of the uterus and double adnexa which was filled with edematous grape-like vesicles.

**Figure 2.**
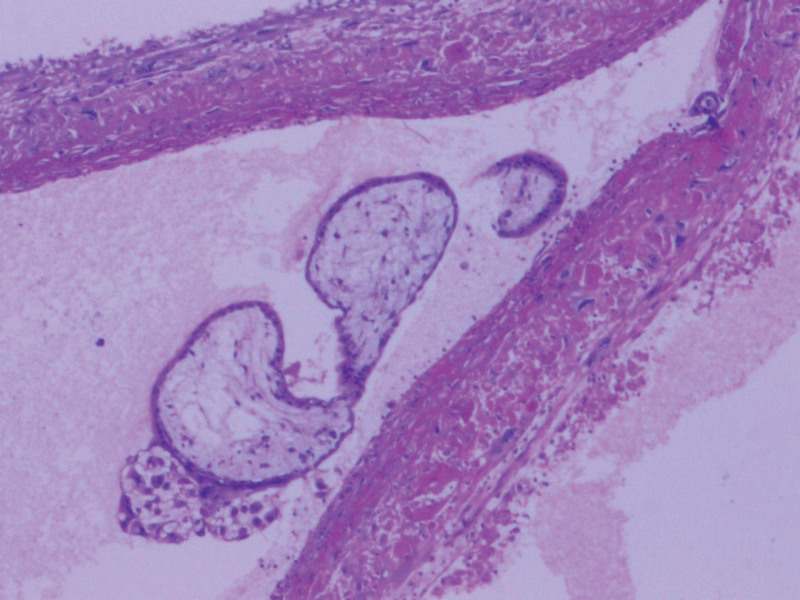
A microscopic image demonstrating a circumferential proliferation of abnormal hyperchromatic trophoblastic cells surrounding edematous hydropic villi (hematoxylin and eosin [H&E] stain).

**Figure 3.**
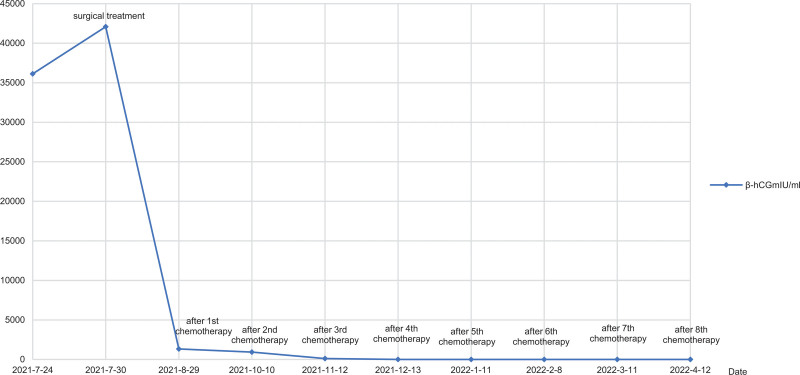
Timeline of serum β human chorionic gonadotropin levels and intervention.

**Figure 4.**
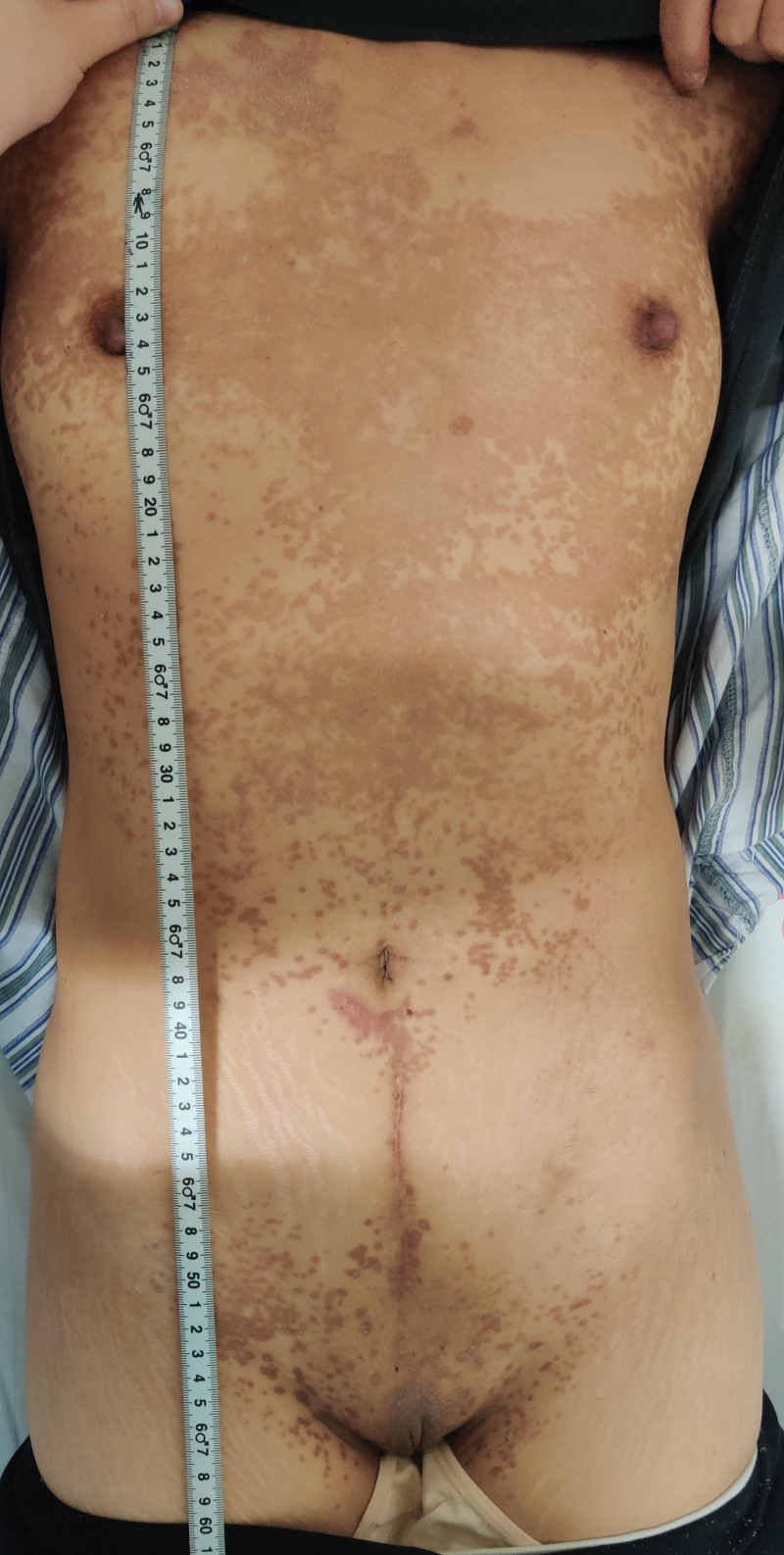
Erythema multiform on the chest, abdomen, groin, monsveneris and labia majora, with edematous edge, desquamative surface, part of them fused.

**Figure 5.**
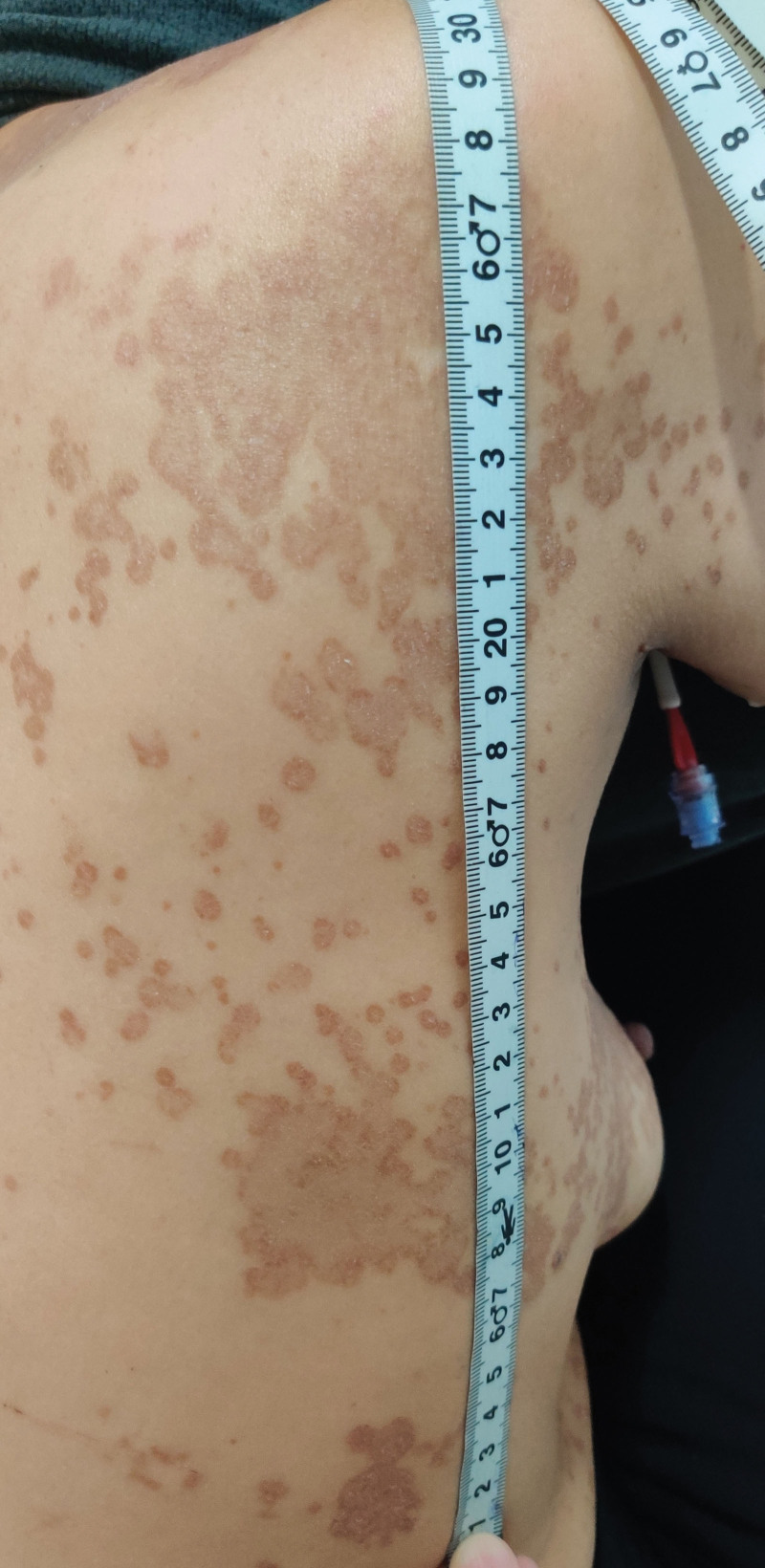
Erythema multiform on the back.

**Figure 6.**
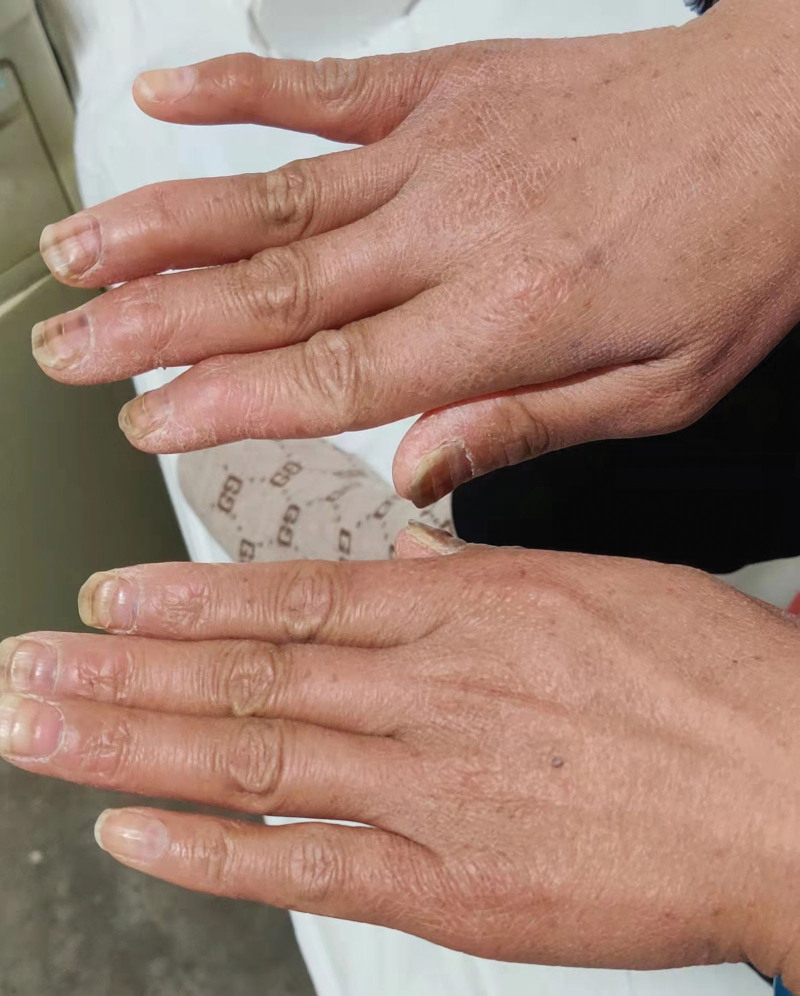
The melanin and transverse sulcus of the fingernails.

In July 2021, a 45-year-old woman patient was admitted to hospital mainly due to colporrhagia. She has regular menstruation cycles and last menstrual period was march 2021 (The exact date is unknown). Four months ago, the volume of last menstrual period was about twice as much as usual, with rotten flesh-like tissue, accompanied by bearing-down pain in lower abdominal, without cough, hemoptysis, headache and dizziness. She was performed curettage twice in local hospital without pathology. Colporrhagia was continuous and increased after sexual intercourse 1 months ago, transvaginal color doppler ultrasound showed gestational trophoblastic disease combined with arteriovenous fistula in left rear wall of uterus. Computed tomography of chest, brain and abdomen were abnormal. Serum β human chorionic gonadotropin (β-hCG) was 36120 mIU/mL. The patient was diagnosed with gestational trophoblastic neoplasia and classified as a stage Ⅰ (International Federation of Gynecology and Obstetrics, 2000) and 4 points (WHO 2000). She received chemotherapy with 5-FU (26–28) mg/(kg*d) and ActD (4–6) mg/(kg*d) in July 27, 2021 to July 29, 2021, then underwent total hysterectomy and bilateral salpingectomy in July 30, 2021. As seen during surgery: the uterus is enlarged as 2 months of pregnancy, with distended left rear wall, uterus and bilateral adnexal were severe congestion. There were a large number of hyperemia vessels in para-uterine tissue and mesentery of fallopian tubes, especially on the left side. There was no ascitic fluid in pelvic cavity. Gross specimen: There were blisters about 0.03 to 0.4 m in diameter in the uterine cavity, myometrium, and left fallopian tube, the right fallopian tube is normal (Fig. 1). Gross specimen: There was 0.4 m nodules in the left rear wall of myometrium, about 0.03m from the serosa. It was consist of 0.05 to 0.1 m yellow-white vesicular tissue, with sparse vascular distribution. Tissue extended downward to the sacral ligament. The upper half of uterine cavity was full of same tissue. Pathology: edema villi can be seen in the uterine cavity, accompanied by obvious proliferation of trophoblast cells, partial degeneration and necrosis, tumor size 0.2*0.15*0.15 m. Invasive mole was diagnosed (Fig. 2). On the 4th day after surgery, she continued chemotherapy. On the 4th day of chemotherapy, the patient has slight nausea and vomiting, no other discomfort. Then she received 4 courses of chemotherapy, the amount of chemotherapy was the same as before, and the serum β-hCG decreased to normal (Fig. 3: timeline of β-hCG levels and intervention). The 6th course of treatment (the first consolidation chemotherapy) was given 5-FU and ActD with an average daily amount of 25 mg/kg and 5.91 ug/kg, respectively. On the 6th day of this course, the patient developed erythema on skin, involving the neck, chest and back, abdomen, groin, monsveneris and labia majora, with edema on the edge of erythema, desquamation on the surface, part of the rash fused and flaky (Figs. 4 and 5). The palms showed flushing and desquamative. Fingernails on both hands can be seen dissolving and transverse sulcus, the melanin is banded from the root of nail to top, but didn’t appear on toenails (Fig. 6). There were no pain and tenderness in above parts. Along with mild nausea, she felt no vomiting, no abdominal pain and diarrhea, and no oral mucosa ulceration. Laboratory tests including blood and urine routine, liver and kidney function, electrolytes and coagulation function were all normal. Erythema multiform (EM) drug eruption was diagnosed by dermatologist, the patient accepted close observation. About 10 days after this course of chemotherapy, the rash was gone and the nail changes returned. Then she continued to receive 2 courses of consolidation chemotherapy, the average daily doses were 5-FU 24.55 mg/kg, Act-D 5.35 ug/kg, and 5-FU 25.46 mg/kg, Act-D 5.56 ug/kg. During 2 course, side response including skin and nail change appeared on 5 to 6th day, vanished on 10th day, as same as before.

## 3. Discussion

Secondary to molar pregnancy, invasive mole mainly refers to mole tissue invading the uterine muscle layer or metastasizing to other parts and continuing to develop, which is a type of gestational trophoblastic neoplasia. The principal treatment consisted of mainly chemotherapy, supplemented by surgery and radiotherapy. Patients are classified as low or high risk based on trophoblastic tumor anatomy staging and modified International Federation of Gynecology and Obstetrics prognostic scoring system. Low-risk type: stage I-III with a score of ≤6, single-agent chemotherapy is preferred; high-risk type: stages I-III and IV with ≥7 points, and combination chemotherapy is recommended. The first-line chemotherapy drugs are methotrexate, ActD, 5-FU, etc.^[8]^

As a thymidine synthase inhibitor, 5-FU is metabolize to 5-FU deoxynucleotide in the body, binds to thymidylate synthase, inhibits thymidylate synthase activity, blocks the methylation of deoxyuridine acid to deoxythymidylate, and affects deoxyribonucleic acid (DNA) synthesis.^[9,10]^ In addition, 5-FU can be converted to 5-FU nucleoside in vivo, which can be incorporated into ribose nucleic acid (RNA) by form of pseudo-metabolites to interfere with protein synthesis, and to inhibit tumor cell growth ultimately.^[11]^ ActD binds to guanine of DNA to form a complex that inhibits RNA polymerases subordinate to DNA and blocks mRNA synthesis. In addition, it can activate intracellular acid endonuclease and related components, promote DNA degradation and induce apoptosis. Activating intracellular signals during the induction of apoptosis, providing a co-stimulatory signal in concert with Fas, it can sensitize Fas antibody-mediated apoptosis-insensitive cell lines to promote apoptosis.^[12,13]^ Chemotherapy combinated with 5-Fu and ActD has been used in clinical practice in China for many years, because of its high cure rate and low cost, but the toxic and side effects have also attracted doctor’s attention in recent years. Its adverse reactions are generally divided into 2 categories: dose-related drug reactions, which are predictable and determined by the characteristics of the drug itself; non-dose-related drug reactions are unpredictable and are related to both individual’s constitution as well as the drug itself. Drug eruptions are mostly the latter.^[14]^

Drug eruption is known as drug-induced dermatitis, refers to the inflammatory response to skin and mucous membranes, caused by the drug being ingested into the human body through oral, injection, inhalation, suppository, perfusion or absorption.^[15]^ Most patients can be cured by immediately stop taking the sensitizing drug and giving antiallergic therapy, however, severe skin drug adverse reactions often occur suddenly, with extensive and severe skin lesions, and may damage the oral mucosa and even severely impair liver and kidney function, causing multiple organ failures and life-threatening.^[16]^

EM is a type of drug eruption that is usually induced by drugs such as anticonvulsants, antibiotics, and allopurinol. Other potential triggers include herpesvirus infection, mycoplasma pneumonia, malignancy, connective tissue disease, and radiation therapy.^[17]^ It is divided into mild and severe. Mild drug eruptions appear as round or oval edematous erythema or papules the size of soybeans to broad beans, with blisters in the center and redness of the edges, often occurring symmetrically. The drug rash with severe erythema multiforme is called SJS or TEN.^[18]^

The onset of SJS/TEN is rapid, and the peripheral symptoms are serious. The edematous erythema and ecchymosis expanded and fused rapidly, accompanied by blisters, bullae and even blood blisters, along with severe pain, high fever, and positive Nissl’s sign. The mucous membranes of the mouth, eyes, vulva and perianal region are red, swollen, erosive, and ruptured. Multiple organs can be involved, such as panophthalmitis, gastrointestinal bleeding, bronchopneumonia, pancreatitis, abnormal liver and kidney function, sepsis and even death.^[19,20]^ Therefore, it is necessary to identify and avoid the development of SJS/TEN early.

Several reasons may explain the mechanism by which 5-FU causes drug eruptions: dihydropyrimidine dehydrogenase is the initial and rate-limiting enzyme of the 5-FU catabolic pathway. It encoded by the dihydropyrimidine dehydrogenase gene (DPYD) gene and responsible for 80% to 90% of the 5-FU clearance,^[21]^ and many studies have shown that decrease in dihydropyrimidine dehydrogenase activity or deficiency in vivo leads to decline in 5-FU clearance, increase in half-life, and thus increased risk of intoxication. In cases of partial or complete deficiency, the 5-FU half-life can be significantly extended from 10 to 15 minutes to 160 minutes or even longer.^[1]^ Until now DPYD has been determined about 40 polymorphic loci, the most commonly toxically associated alleles of the DPYD gene are DPYD*2A (about 50% of the total mutations), DPYD*9B, DPYD*13 and hab B3.^[20]^ In addition to DPYD, polymorphisms in other genes such as thymidylate synthase, methylenetetrahydrofolate reductase, enolase superfamily members 1, and cytidine deaminase have also been associated with fluorouracil toxicity.^[22–25]^ Sex difference also leads to different clearance of 5-FU, and higher toxicity in females.^[26]^ Women’s body mass index is generally smaller than men, and the fat content is higher, which result in smaller percentage of metabolically active fat-free body weight. But the dose of chemotherapy calculated by body weight does not take into account the sex difference of fat-free body weight.^[27,28]^ In addition to sex, advanced age and renal impairment are also be concerned with fluorouracil poisoning, the reasons are unclear.^[28]^ Ultraviolet b (UVB) is also involved in the formation of EM: fluorouracil has been reported to cause photosensitive rashes such as sunburn reactions and light distribution pigmentation.^[29]^ Under UVB irradiation, fluorouracil induces photooxidation of superoxide anions and proteins, triggering cell damage.^[30]^ Total 15% of white patients develop erythema a few days after UVB radiation.^[31]^ Yoshimasu et al showed that t-cell receptor-α chain knockout mice develop cutaneous lupus erythematosus under the action of 5-FU and ultraviolet light,^[32]^ and speculated the similar mechanism in EM. UVB also effectively stimulates keratinocytes to release cytokines, including Interleukin-1, tumor necrosis factor-α and interleukin-6, serotonin, prostaglandins, lysosomal enzymes and kinins. These pro-inflammatory factors directly or indirectly induces abnormal expression of certain adherent molecules such as intercellular adhesion molecules on keratinocytes, and induces cell damage.^[33]^ Degenerative changes in sweat glands have been found in patients receiving 5-FU therapy, resulting in a decrease in the sweat glands excretion of uric acid, which has a protective effect on human skin exposed to sunlight.^[34]^ 5-FU also can causes excess production of reactive oxygen radicals and inflammatory mediators, triggering cell damage,^[7]^ and 5-FU damages immature keratinocytes, stem cells, and the basal cell layer of the epithelium, resulting in a loss of epithelial renewal capacity, and abnormally developing keratinocytes to be more sensitive to cytotoxic drugs and UVB.

In addition to trophoblastic disease, EM has been reported in patients receiving 5-FU chemotherapy for other types of cancer. Jung Hyun Han^[17]^ et al reported a 54-year-old male patient with rectal cancer who received 3 cycles of 5-FU chemotherapy, followed by 180 cGy/day radiotherapy. Thirteen days after the fourth cycle of chemotherapy (at the end of the 26th radiotherapy), erythema-like papules and fused macules spread from the buttock to the hand, back and ear. Histopathology of skin biopsy of the buttocks revealed vacuole-like changes in the basal layer, mild inflammatory infiltration around the blood vessels, and the presence of many eosinophils in the dermis, which was diagnosed as EM. The lesions resolved after 2 weeks of treatment. Then he received next cycle of chemotherapy with the same regimen, and 13 days later, EM recurred on the arms and legs and the lesions resolved with antihistamine and steroid treatments. Except for the site described in this case, erythema can also occur in other sites, such as the scrotum: a 70-year-old man had a painful, burning and itchier rash on the scrotum that appeared 8 days after he started using 5% 5-FU cream to treat actinic keratosis twice daily.^[35]^ Scrotal skin aspiration biopsy histopathology shows interfacial dermatitis with keratosis, epidermal atrophy, interfacial vacuoles, and occasionally basal keratinocyte necrosis. The rash was subsidence by taking prednisone and using betamethasone topicality for 2 weeks. Seborrheic dermatitis and serpentine supravenous pigmentation due to fluorouracil drugs were reported too.^[36,37]^ The clinical manifestations of above cases were mild and cured after drug withdrawal and anti-allergy treatment, but the rash will progress and lead to more serious skin reactions, characterized by erythema of skin all over body, with dry eyes and tears, and oral cavity mucous membrane inflammation, extensive epidermal denudation, positive Nissl sign, namely SJS/TEN.^[38]^ General SJS/TEN can be relieved after treatment with glucocorticoids and immunoglobulin, but it will be life-threatening when the disease is serious or not timely diagnosed and treated. Kavya Karthikeyan et al^[39]^ reported a serious case of SJS. A 70-year-old male patient was diagnosed with adenocarcinoma of the pancreas, stage 4 and started on chemotherapy with Capecitabine. The patient presented with skin erosions and hyperpigmentation, excoriation in the scrotal region, bleeding mucositis, vomiting and loose stools. Along with blood routine altered (hemoglobin: 8.3 g/dL, platelets: 15,000/mcL), liver function became abnormal (total bilirubin: 1.3 mg/dL, direct bilirubin: 0.7 mg/dL), blood glucose level was highly elevated (with a general random blood glucose level of 372 mg/dL–460 mg/dL). The vital signs was blood pressure 160/90 mm Hg. He was considered to Capecitabine induced SJS. The patient was discontinued Capecitabine, injected dexamethasone, applied 2% mupirocin ointment and alovit gel for skin and scrotal lesions, used Triamcinolone for oral ulcers. He was treated with intravenous fluids and antibiotics (changed from Amikacin and Piperacillin Tazobactam to Meropenem Sulbactam and Metronidazole) because of the progressive and severe bleeding from the mouth and groin area. Throat swab culture and sensitivity indicated the heavy growth of Candida albicans. Because of discoloration and discomfort in eyes, he was concluded as conjunctival hemorrhage with erosion and peeling of the conjunctiva. Then he accepted Fluconazole injection, Ofloxacin and Hydroxypropylmethylcellulose eye drops. His hemoglobin and platelevels decreased day by day, but he didn’t respond to infusion of red blood cells, platelets concentrate, and fresh frozen plasma. At last, the kidney functional deteriorated quickly, the patient was died eventually. Regarding chemotherapy-induced nail changes, some experts think that nail stromal cells make nails easy targets for anti-mitotic activity of chemotherapy drugs. Hyperpigmentation of nails is the most common nail change, which is the result of activation of stromal melanocytes.^[40]^ Vera Teixeira^[41]^ et al reported a 48-year-old woman who developed acral hyperpigmentation during tegafur intake for advanced rectal adenocarcinoma stage C, Involving the nails, which was similar to our case.

The etiology of ActD drug eruptions is concentrated on eccrine squamous duct metaplasia. Two children with rhabdomyosarcoma who received ActD chemotherapy developed lesions in the axillary, groin, and central venous catheter outlet, and skin biopsies showed dermatitis and sweat angiitis metaplasia.^[42]^ A 15-year-old boy received chemotherapy with ActD for left paratesticular embryonic rhabdomyosarcoma and developed an persistent serpentine supravenous pigmentation rash on the left forearm. Skin biopsy showed mixed inflammatory infiltration of lymphohistiocytes and polymorphonuclear cells around apocrine glands with squamous metaplasia, and extensive epithelial necrosis and vacuolation in eccrine glands, which were consistent with the cytotoxic response associated with eccrine neutrophil hidradenitis.^[43]^ ActD can also lead to hand-foot syndrome which show as the skin of the hands and/or feet burning, erythema, swelling, blister and chapped. ActD can also cause TEN: a 2-year-old boy was received combination chemotherapy with dactinomycin and vincristine because of left kidney nephroblastoma. He developed multiple morbilliform rashes on his limbs and chest, which rapidly progressed to necrotic rashes and confluence erythematous with blisters and was diagnosed as TEN. The skin biopsy showed loss of epidermis secondary to full thickness necrosis, with mild chronic inflammation noted in the dermo-epidermal junction and upper dermis.^[44,45]^

In summary, we reported a case of EM after 5-FU and ActD chemotherapy, with skin and nail changes. The etiological association between the drug and the adverse effects has been demonstrated by repeated use and discontinuation of chemotherapy agents. Because the patient did not undergo allergen and related etiology testing, anaphylaxis caused by a single drug or a combination of drugs could unable to determine. At present, DPYD gene detection has not been performed in most cases of skin adverse reactions induced by 5-FU. The possible reason is that most rashes can be improved by suspected drug withdrawal, topical and oral drug administration. But the increased toxicity of 5-FU caused by DPYD gene deficiency has been confirmed. If the rash continues to progress, it will develop into SJS/TEN, which is life-threatening in severe cases. Early recognition should be made to prevent the progression of the disease. Therefore, when applying 5-FU and ActD or other chemotherapeutic drugs, we should pay attention to the dosage of the drug, monitor adverse reactions strictly, avoid sun exposure, or use sun-protective clothing and sunscreen.^[29]^ Once severe drug eruptions are suspected or diagnosed, allergenic drugs should be discontinued immediately. At the same time, the blood routine, urine routine, erythrocyte sedimentation rate, liver and kidney function, electrolyte and immune tests, skin biopsy, and DPYD gene testing should be performed. It should be emphasized that soluble Fas ligand, granolysin, granzyme B and perforin are markers of SJS/TEN which may lead to serious condition.^[46]^ Treatment includes debridement, keeping the wound moist and clean to reduce fluid loss and prevent infections, using of oral or intravenous fluids and nutritional support, empiric antibiotic therapy to reduce the risk of infection, glucocorticoid and immunoglobulin therapy, and plasma exchange is feasible when necessary.^[47–50]^

## Author contributions

**Data curation:** Tengfei Li, Yuan Wang.

**Formal analysis:** Yuan Wang, Xiaoguang Zhang.

**Investigation:** Mengdi Wang, Yibin Liu.

**Resources:** Shan Wang,Yuan Wang.

**Supervision:** Lijuan Zhang.

**Validation:** Lijuan Zhang.

**Writing – original draft:** Shan Wang, Tengfei Li.

**Writing – review & editing:** Shan Wang, Lijuan Zhang.
